# Lowered cognitive function and the risk of the first events of cardiovascular diseases: findings from a cohort study in Lithuania

**DOI:** 10.1186/s12889-021-10843-4

**Published:** 2021-04-24

**Authors:** Dalia Luksiene, Laura Sapranaviciute-Zabazlajeva, Abdonas Tamosiunas, Ricardas Radisauskas, Martin Bobak

**Affiliations:** 1 Laboratory of Population Studies of the Institute of Cardiology, Medical Academy, Lithuanian University of Health Sciences, LT-50162 Kaunas, Lithuania; 2 Department of Environmental and Occupational Medicine, Faculty of Public Health, Medical Academy, Lithuanian University of Health Sciences, LT-47181 Kaunas, Lithuania; 3 Department of Health Psychology, Lithuanian University of Health Sciences, LT-47181 Kaunas, Lithuania; 4 Department of Preventive Medicine, Faculty of Public Health, Medical Academy, Lithuanian University of Health Sciences, LT-47181 Kaunas, Lithuania; 5 Department of Epidemiology and Public Health, University College London, London, WC1E 6BT UK

**Keywords:** Cognitive functions, Risk of first cardiovascular disease event, Cardiovascular mortality, Cohort study

## Abstract

**Background:**

The purpose of this prospective cohort study was to examine whether the level of cognitive function at the baseline expressed as a cognitive function composite score and score of specific domains predict the risk of first cardiovascular disease (CVD) events in middle-aged and older populations.

**Methods:**

Seven thousand eighty-seven participants, men and women aged 45–72 years, were assessed in the baseline survey of the Health Alcohol Psychosocial Factors in Eastern Europe (HAPIEE) study in 2006–2008 in the city of Kaunas, Lithuania. During 10 years of follow-up, the risk of first non-fatal events of CVD and death from CVD (excluding those participants with a documented history of CVD and/or ischemic heart disease (IHD) diagnosed at the baseline survey) was evaluated. Cox proportional hazards regression models were applied to examine how cognitive function predicts the first events of CVD.

**Results:**

During the follow-up, there were 156 deaths from CVD (49 women and 107 men) and 464 first non-fatal CVD events (195 women and 269 men) registered. The total number of first CVD events was 620 (11.5%). After adjustment for sociodemographic factors, biological and lifestyle risk factors and illnesses, a decrease per 1 standard deviation in different cognitive function scores significantly increased the risk of a first event of CVD (immediate verbal recall score - by 17% in men and 32% in women; delayed verbal recall score – by 17% in men and 24% in women; and a composite score of cognitive function – by 15% in men and 29% in women). Kaplan-Meier survival curves for the probability of a first cardiovascular event according to the categories of a composite score of cognitive function, revealed that a lowered cognitive function predicts a higher probability of the events compared to normal cognitive function (*p* < 0.05).

**Conclusions:**

The findings of this follow-up study suggest that men and women with lower cognitive functions have an increased risk for a first event of CVD compared to participants with a higher level of cognitive functions.

## Background

The introduction is long, and it is not clear which is the added value of the present study.

Several epidemiological studies have demonstrated a relationship between the level of cognitive function and the incidence of some chronic conditions such as hypertension, ischemic heart disease (IHD), diabetes, and stroke [1–4]. As well as with the risk of mortality: both from all – causes of death and specific causes of death – cardiovascular diseases (CVD) (ischemic heart disease (IHD) and stroke) and other chronic diseases [5–8]. The majority of studies have shown that cognitive function determined at the baseline is inversely associated with a risk of the incidence and mortality from mentioned chronic non-communicable diseases. The association is found to be independent of traditional cardiovascular risk factors [9, 10]. Cognitive impairment and dementia, the same as chronic diseases such as CVD and diabetes, are becoming highly prevalent among the ageing of populations and carry a huge personal and economic burden [11]. Similar vascular pathology caused mainly by the atherosclerotic process is linking both cognitive impairment and chronic diseases such as IHD and stroke [12, 13]. Although the relationship between lowered cognitive function, and CVD incidence or mortality from CVD is quite widely covered by epidemiological studies. Most studies analyse only the risk of stroke incidence and mortality from a stroke [4, 14, 15]. Less is known regarding the link between cognitive function, especially having in mind specific domains of cognitive function, and the incidence of the first event of CVD including both non-fatal and fatal cases of IHD. A small number of epidemiological studies had included the assessment of the risk of first events of CVD according to the level of cognitive function at the baseline [16].

The aim of this prospective cohort study, therefore, was to examine whether the level of cognitive function at the baseline expressed as a cognitive function composite score and the score of specific domains predict the risk of a first CVD event in middle-aged and older populations.

## Methods

This prospective cohort study is part of the international project Health, Alcohol and Psychosocial Factors in Eastern Europe (HAPIEE) [17]. The baseline survey was conducted between 2006 and 2008 in the city of Kaunas, Lithuania. The examined cohort was followed up for the first event of CVD until January 1, 2017.

### Study population

The baseline survey recruited 7087 (response rate 64.8%) men and women in Kaunas aged 45–72 years, of which 1692 individuals were excluded for the following reasons: did not complete all cognitive tests or had incomplete information on other study variables (*n* = 183), had a history of CVD (previous stroke and/or history of myocardial infarction) (*n* = 1509). Therefore, the analytical sample of this study comprised 5395 participants with complete baseline data.

Ethical consent was obtained from the Ethics Committee at University College London, UK, and from the Kaunas Regional Biomedical Research Ethics Committee, Lithuania (January 11, 2005; No. 05/09) and informed consent was obtained from all participants.

### Cognitive function assessment

All participants underwent a battery of five cognitive tests: immediate and delayed verbal memory, semantic verbal fluency, speed and concentration and numerical ability. Verbal memory was assessed by testing the immediate and delayed recall of 10 words. Immediate and delayed recall scores ranged from 0 to 10. An animal fluency test was used to assess semantic verbal fluency. Participants were asked to name as many animals as possible for 1 min. The score of semantic verbal fluency is equal to the total number of animals remembered correctly (excluding repeated names of animals and non-animals). Speed and concentration were tested by asking the participants to cross out as many letters P and W as possible within 1 min, using a sheet containing random letters of the alphabet. Numerical ability was assessed using four questions involving simple calculations based on everyday life situations. The number of correct responses to the questions comprised the numeracy score (in the range 0 to 4). The details of cognitive assessment have been published previously [18].

Higher scores of verbal memory, numerical ability, semantic verbal fluency, speed and concentration tests indicate better cognitive function.

We calculated a composite score of cognitive function by averaging z-scores for each test and summing the results. The participants who scored one standard deviation (SD) or more below their age and education specific means of the composite score of cognitive function have been assigned into the lowered cognitive function group.

### Covariates

The covariates and their classification were presented in detail in our previous publications [8, 18]. These covariates included age, gender, education, marital status, depressive symptoms, psychological well-being (PWB), lifestyle (smoking status, physical activity during leisure time, and alcohol consumption), and medical history (previous myocardial infarction, stroke and diabetes mellitus) determined using a standard questionnaire. We also included into the statistical analysis as covariate variables laboratory analyses (total cholesterol, low-density lipoprotein (LDL) cholesterol, high-density lipoprotein (HDL) cholesterol, triglyceride, and fasting glucose levels) and measurements (arterial blood pressure, body weight and height, waist circumference). Definitions of the health conditions (arterial hypertension, IHD and stroke) have been also described in our previous article [18].

### Registration of the first cardiovascular events

The study participants were followed up from the beginning of the baseline survey date until January 1, 2017. The cohort study outcomes were measured as the first non-fatal events of CVD and cases of death from CVD (excluding those participants with a documented history of CVD and/or IHD diagnosed at the baseline survey). Non-fatal events of CVD included the first events of IHD (unstable angina pectoris, possible and definite acute myocardial infarction according to the criteria of the Multinational Monitoring of Trends and Determinants in Cardiovascular Disease (MONICA) project) and stroke (according to the criteria of the MONICA project) [19]. Non-fatal CVD events were collected from Kaunas’ Ischemic Heart Disease and Stroke Registers. Data from the Kaunas Mortality Register based on death certificates were used for the registration of death events in the study participants. Causes of death were coded by the International Classification of Diseases (ICD) (version 10). Mortality from CVD included death from IHD, stroke and other vascular diseases (ICD codes I00 – I99).

### Statistical analysis

We compared the baseline descriptive characteristics (means and standard errors (SE) – for continuous variables and proportions in percentages – for categorical variables) in three groups of men and women at the end of the follow-up: alive and without an event of CVD, with a first event of CVD, and dead from causes other than CVD. The differences in the age-adjusted means of variables between groups were tested using T-test and ANOVA analysis with Bonferroni multiple comparison tests. We used a chi-squared test and z test with Bonferroni corrections for comparing the differences in proportions. The difference was statistically significant when *p* < 0.05. We generated the Kaplan-Meier plots separately for men and women for assessing the cumulative risk of the first CVD event according to the categories of a composite score of cognitive function (normal and lowered) at the baseline. A long-rank test was applied to compare the difference between cognitive function categories. We fitted Cox proportional hazards regression models to examine whether specific cognitive function domains at the baseline and the composite score of cognitive function predict the first event of CVD. Hazard ratios (HR) and corresponding 95% confidence intervals (CI) were calculated. The risk of a first event of CVD was calculated per each one SD decrease of a composite score of cognitive function and five other cognitive function tests. For multivariate analysis, we entered all variables that were significantly associated with the risk of a first event of CVD in the univariate analysis. Several models were fitted separately for men and women. Model 1 was adjusted for education, marital status (categorical), and age (continuous). Model 2 was adjusted for all variables in Model 2 plus smoking (categorical), physical activity during leisure time, alcohol consumption, systolic or diastolic blood pressure, total cholesterol, HDL cholesterol, LDL cholesterol, triglycerides, fasting glucose and body mass index (BMI) (all continuous). Model 3 was adjusted for all variables in Model 2, plus depression symptoms (categorical) and PWB (continuous), and existing illness – diabetes mellitus (categorical). All statistical analyses were carried out using IBM SPSS statistics 20.0 software.

## Results

The mean duration and SD of the follow-up of the participants were 9.10 ± 1.79 years among women and 8.50 ± 2.40 years among men. During the follow-up, there were 156 deaths from CVD (49 women and 107 men) and 464 first non-fatal CVD events (195 women and 269 men) registered. The total number of first CVD events was 620 (11.5%).

The characteristics of the respondents at the baseline survey according to the first event of CVD are presented in Table 1 and Table 2. Men and women who had their first event of CVD during the follow-up period were older, less educated and had a higher proportion of widowers at the baseline survey than those alive and without CVD events at the end of the follow-up. During the initial study, the age-adjusted means of some biologic factors, such as systolic and diastolic blood pressure, triglycerides, the fasting glucose level had been higher and the HDL cholesterol level had been lower in men and women who had their first event of CVD compared to those who were alive and without CVD events during the follow-up. Moreover, it was determined that the respondents who had their first event of CVD at the follow-up had been more often diagnosed with diabetes mellitus, arterial hypertension and obesity at the baseline survey than those who did not have any CVD events. Meanwhile, men who had their first event of CVD at the follow-up had been smokers more often during the initial study than those without any CVD events during the follow-up study. The men and women who had their first event of CVD during the follow-up had lower PWB at the baseline survey more often compared to those without CVD. Men who had their first event of CVD during the follow-up more often had depressive symptoms at the baseline compared to those without CVD event at the follow-up.

**Table 1:** Baseline characteristics by survival status of men in the Kaunas HAPIEE study (2006–2008)

Variables	Living status			*P* from Anova
	Alive and no non-fatal CVD event (*n* = 2042)	1st CVD event (*n* = 343)	Dead from other than CVD causes of death (*n* = 175)	
Age, years	55.4 ± 0.17	59.2 ± 0.44 ^b^	60.3 ± 0.51 ^b^	< 0.001
Immediate verbal recall sum^a^, score	22.1 ± 0.08	20.3 ± 0.22 ^b^	20.2 ± 0.30 ^b^	< 0.001
Delayed verbal recall^a^, score	7.82 ± 0.04	7.10 ± 0.10 ^b^	6.96 ± 0.14^b^	< 0.001
Semantic verbal fluency^a^	22.8 ± 0.14	21.3 ± 0.31^b^	21.0 ± 0.47^b^	< 0.001
Numerical ability^a^, score	3.15 ± 0.02	3.00 ± 0.04 ^b^	3.04 ± 0.06	< 0.001
Cognitive speed and attention^a^	16.6 ± 0.10	15.3 ± 0.26 ^b^	14.4 ± 0.35 ^b^	< 0.001
Composite score of cognitive function^a^	−0.06 ± 0.01	−0.22 ± 0.03 ^b^	−0.18 ± 0.05	< 0.001
Systolic blood pressure^a^, mm Hg	142.0 ± 0.43	149.3 ± 1.20 ^b^	145.6 ± 1.64	< 0.001
Diastolic blood pressure^a^, mm Hg	91.9 ± 0.27	95.4 ± 0.74 ^b,^	92.9 ± 0.99 ^c^	< 0.001
Total cholesterol^a^, mmol/L	5.82 ± 0.02	5.92 ± 0.06	5.56 ± 0.09^b, c^	0.002
HDL cholesterol^a^, mmol/L	1.43 ± 0.01	1.36 ± 0.02 ^b^	1.50 ± 0.03 ^b, c^	< 0.001
LDL cholesterol^a^, mmol/L	3.73 ± 0.02	3.81 ± 0.06	3.46 ± 0.08 ^b, c^	0.001
Triglyceride^a^, mmol/L	1.47 ± 0.02	1.72 ± 0.07 ^b^	1.29 ± 0.05 ^c^	< 0.001
Fasting blood glucose^a^, mmol/L	5.67 ± 0.02	5.88 ± 0.08 ^b,^	5.78 ± 0.09	0.008
Body mass index^a^, kg/m^2^	28.0 ± 0.09	29.1 ± 0.25 ^b^	27.1 ± 0.38 ^b, c^	< 0.001
Physical activity in leisure time^a^, hours/week	17.1 ± 0.26	17.5 ± 0.72	16.8 ± 1.05	0.783
Absolute alcohol^a^, drinks/week	60.7 ± 2.44	76.4 ± 9.51	62.1 ± 9.99	0.09
PWB^a^, score	39.9 ± 0.12	39.1 ± 0.31 ^b^	37.5 ± 0.45 ^b, c^	< 0.001
				*P* from χ^2^
Diabetes mellitus % (*n*)	5.2 (104)	8.8 (29) ^b^	10.8 (18) ^b^	0.001
Arterial hypertension % (*n*)	66.6 (1360)	77.8 (266) ^b^	75.0 (129)	< 0.001
Body mass index, % (*n*)				< 0.001
< 25.0 kg/m2	24.6 (503)	18.4 (99)^b^	31.8 (56) ^c^	
25.0–29.9 kg/m2	46.3 (945)	42.0 (144)	43.2 (76)	
> = 30.0 kg/m2	29.1 (594)	39.7 (136) ^b^	25.0 (44) ^c^	
Smoking habits % (*n*)				< 0.001
Smokers	35.4 (722)	41.1 (141)	45.4 (79) ^b^	
Former smokers	27.0 (551)	30.9 (106)^b^	28.7 (50)	
Never smokers	37.7 (769)	28.0 (96) ^b^	25.9 (45) ^b^	
Marital status % (*n*)				0.003
Single	2.0 (41)	2.6 (9)	1.7 (3)	
Married	84.8 (1732)	80.5 (276)	76.0 (133) ^b^	
Co-habiting	2.2 (44)	1.5 (5)	2.3 (4)	
Divorced	8.0 (164)	9.0 (31)	12.6 (22)	
Widowed	3.0 (61)	6.4 (22) ^b^	7.4 (13)^b^	
Education % (*n*)				< 0.001
Primary	2.7 (56)	7.3 (25) ^b^	8.0 (14) ^b^	
Vocational	6.6 (135)	13.1 (45) ^b^	16.6 (29) ^b^	
Secondary	33.9 (693)	34.1 (117)	35.4 (62)	
College	19.9 (407)	17.5 (60)	19.4 (34)	
University	36.8 (751)	28.0 (96) ^b^	20.6 (36) ^b^	
Depression scale score % (*n*)				< 0.001
> = 4	13.0 (260)	18.6 (63) ^b^	22.2 (37) ^b^	
< 4	87.0 (1742)	81.4 (276) ^b^	77.8 (130) ^b^	
Cognitive function % (*n*)				0.025
Normal	82.4 (1683)	77.3 (265)	77.1 (135)	
Lowered	17.6 (359)	22.7 (78)	22.9 (40)	
PWB % (*n*)				< 0.001
Higher	57.6 (1089)	52.2 (167)	39.8 (64) ^b, c^	
Lower	42.4 (800)	47.8 (153)	60.2 (97) ^b, c^	

^a^ Age-adjusted means and standard errors. ^b^ *p* < 0.05 compared to alive and no non-fatal CVD event group, Bonferroni test, ^c^ *p* < 0.05 compared to 1st CVD event group, Bonferroni test

*CVD* cardiovascular diseases, *HAPIEE* Health, Alcohol and Psychosocial factors In Eastern Europe, *HDL* high-density lipoprotein, *LDL* low-density lipoprotein, *PWB* psychological well-being, *SE* standard error

**Table 2:** Baseline characteristics by survival status of women in the baseline survey of the Kaunas HAPIEE study (2006–2008)

Variables	Living status			*P* from Anova
	Alive and no non-fatal CVD event (*n* = 2762)	1st CVD event (*n* = 198)	Dead from other than CVD causes of death (*n* = 124)	
Age, years	55.5 ± 0.14	61.5 ± 0.56 ^b^	58.9 ± 0.77 ^b, c^	< 0.001
Immediate verbal recall sum^a^, score	23.5 ± 0.07	21.8 ± 0.28 ^b^	22.7 ± 0.34 ^b^	< 0.001
Delayed verbal recall^a^, score	8.40 ± 0.03	7.69 ± 0.13 ^b^	7.90 ± 0.14^b^	< 0.001
Semantic verbal fluency^a^	22.7 ± 0.12	20.5 ± 0.46^b^	21.4 ± 0.63	< 0.001
Numerical ability^a^, score	2.92 ± 0.01	2.82 ± 0.05	2.78 ± 0.06	0.028
Cognitive speed and attention^a^	17.9 ± 0.09	16.1 ± 0.35 ^b^	15.7 ± 0.41 ^b^	< 0.001
Composite score of cognitive function^a^	0.11 ± 0.01	0.02 ± 0.04	0.03 ± 0.05	0.032
Systolic blood pressure^a^, mm Hg	131.6 ± 0.37	142.1 ± 1.54 ^b,^	134.0 ± 1.83 ^c^	< 0.001
Diastolic blood pressure^a^, mm Hg	86.6 ± 0.21	91.0 ± 0.89 ^b,^	87.5 ± 1.02 ^c^	< 0.001
Total cholesterol^a^, mmol/L	6.04 ± 0.02	6.08 ± 0.08	5.99 ± 0.10	0.791
HDL cholesterol^a^, mmol/L	1.63 ± 0.01	1.47 ± 0.03 ^b^	1.60 ± 0.04^c^	< 0.001
LDL cholesterol^a^, mmol/L	3.79 ± 0.02	3.87 ± 0.07	3.76 ± 0.09	0.562
Triglyceride^a^, mmol/L	1.35 ± 0.02	1.62 ± 0.07 ^b,^	1.33 ± 0.06 ^c^	< 0.001
Fasting blood glucose^a^, mmol/L	5.71 ± 0.02	5.95 ± 0.10 ^b^	5.82 ± 0.10	0.004
Body mass index^a^, kg/m^2^	28.8 ± 0.11	31.6 ± 0.45 ^b^	30.3 ± 0.54 ^b^	< 0.001
Physical activity in leisure time^a^, hours/week	19.7 ± 0.21	20.4 ± 0.92	17.1 ± 0.87	0.025
Absolute alcohol^a^, drinks/week	21.0 ± 0.63	15.1 ± 1.84 ^b^	19.8 ± 3.12	0.043
PWB^a^, score	38.4 ± 0.11	37.1 ± 0.50 ^b^	37.1 ± 0.59 ^b^	0.001
				P from χ^2^
Diabetes mellitus % (*n*)	5.1 (137)	14.8 (29) ^b^	9.2 (11)^b^	< 0.001
Arterial hypertension % (*n*)	51.5 (1417)	74.7 (148) ^b,^	59.7 (74) ^c^	< 0.001
Body mass index, % (*n*)				< 0.001
< 25.0 kg/m2	26.6 (735)	15.7 (31) ^b^	16.9 (21) ^b^	
25.0–29.9 kg/m2	37.0 (1020)	26.9 (53) ^b^	39.5 (49)	
> = 30.0 kg/m2	36.4 (1005)	57.4 (113) ^b^	43.5 (54) ^c^	
Smoking habits % (*n*)				0.036
Smokers	14.9 (411)	10.6 (21)^b^	13.7 (17)	
Former smokers	8.8 (243)	7.1 (14)	15.3 (19)^b^	
Never smokers	76.3 (2107)	82.3 (163)	71.0 (88)	
Marital status % (*n*)				0.004
Single	6.3 (174)	6.0 (12)	7.3 (9)	
Married	60.8 (1678)	54.8 (109)	50.0 (62) ^b^	
Co-habiting	1.3 (35)	0.0 (0)	1.6 (2)	
Divorced	18.0 (496)	15.6 (31)	21.0 (26)	
Widowed	13.7 (378)	23.6 (47) ^b^	20.2 (25)	
Education % (*n*)				< 0.001
Primary	2.8 (78)	8.6 (17) ^b^	7.3 (9) ^b^	
Vocational	5.4 (148)	8.1 (16)	10.6 (13) ^b^	
Secondary	25.7 (709)	27.3 (54)	23.6 (29)	
College	28.7 (792)	32.3 (64)	29.3 (36)	
University	37.5 (1035)	23.7 (47) ^b^	29.3 (36)	
Depression scale score % (*n*)				0.129
> = 4	26.2 (711)	32.7 (64)	28.6 (34)	
< 4	73.8 (2002)	67.3 (132)	71.4 (85)	
Cognitive function % (*n*)				0.048
Normal	89.3 (2467)	84.3 (167)	85.5 (106)	
Lowered	10.7 (295)	15.7 (31)	14.5 (18)	
PWB % (*n*)				0.005
Higher	60.6 (1552)	51.1 (91) ^b^	50.0 (57)	
Lower	39.4 (1010)	48.9 (87) ^b^	50.0 (57)	

^a^ Age-adjusted means and standard errors. ^b^ p < 0.05 compared to alive and no non-fatal CVD event group, Bonferroni test, ^c^ *p* < 0.05 compared to 1st CVD event group, Bonferroni test

*CVD* cardiovascular diseases, *HAPIEE* Health, Alcohol and Psychosocial factors In Eastern Europe, *HDL* high-density lipoprotein, *LDL* low-density lipoprotein, *PWB* psychological well-being, *SE* standard error

Age-adjusted variables of cognitive function among men and women who had their first event of CVD, such as immediate verbal recall, delayed verbal recall, semantic verbal fluency, cognitive speed and attention were significantly lower than among the alive and without any CVD events individuals at the end of the follow-up. However, age-adjusted numerical ability and a composite score of cognitive function differed only in the group of men.

**Fig. 1:**
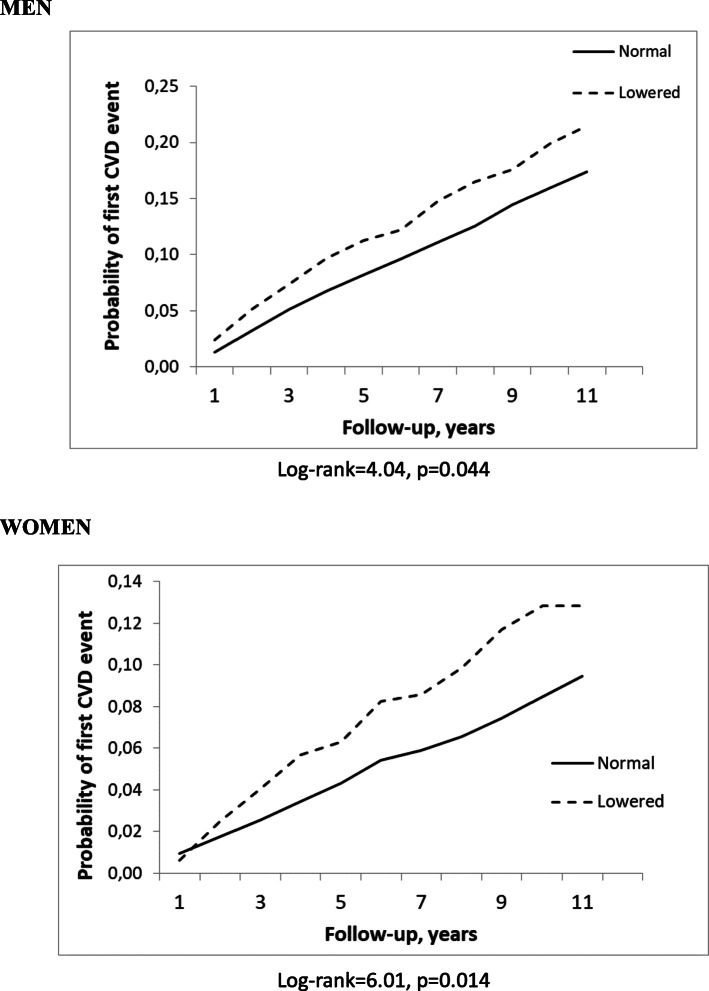
Kaplan-Meier probability curves for the first cardiovascular event according to the categories of a composite score of cognitive function for men and women, adjusted for age

Kaplan-Meier probability curves for the first cardiovascular event according to the categories of a composite score of cognitive function (normal and lowered) for men and women, adjusted for age, are presented in Fig. 1. The log-ranked test revealed that the cumulative probability of a first event of CVD within 10 years of follow-up significantly differs (*p* < 0.05) for two levels of the composite score of cognitive function. The lowered composite cognitive function score predicts a higher probability of a first event of CVD compared with normal cognitive function (among men and women the probability rates were 18.1, and 11.7%, respectively).

Table 3 presents the risk of a first event of CVD in men, and Table 4 presents the risk of a first event of CVD in women after adjustment for socio-demographic, lifestyle, and biological risk factors, existing illness, depressive symptoms, and PWB. We evaluated the risk for the first event of CVD concerning the scores of various cognitive functions.

**Table 3:** Risk of a first event of CVD^a^ by lower cognitive function levels^b^ in men, Kaunas HAPIEE study, 2006–2016

Cognitive function	Cox models					
	Model 1		Model 2		Model 3	
	HR	95% CI	HR	95% CI	HR	95% CI
Immediate verbal recall sum	1.21	1.09–1.34	1.17	1.05–1.31	1.17	1.04–1.32
Delayed verbal recall	1.19	1.07–1.32	1.17	1.05–1.30	1.17	1.05–1.32
Semantic verbal fluency	1.07	0.96–1.20	1.08	1.05–1.10	1.03	0.91–1.17
Numerical ability	1.07	0.96–1.20	1.04	0.93–1.18	1.01	0.89–1.14
Cognitive speed and attention	1.10	0.98–1.24	1.07	0.95–1.22	1.04	0.91–1.18
Composite score of cognitive function	1.09	1.07–1.12	1.08	1.06–1.11	1.15	1.03–1.28

^a^ Individuals with existing CVD (IHD and stroke) at baseline survey were excluded, ^b^ per each 1 standard deviation decrease

*HAPIEE* Health, Alcohol and Psychosocial factors In Eastern Europe, *HR* hazard ratios, *CI* confidence interval

Model 1 adjusted for age (continuous variable), education and marital status (categorical variables)

Model 2 adjusted for all the variables in Model 1 plus lifestyle (smoking – categorical, physical activity in leisure (continuous – hours/week), alcohol consumption (continuous – drinks/week) and biologic factors (systolic or diastolic blood pressure – continuous, total cholesterol, HDL cholesterol, LDL cholesterol, triglycerides, fasting glucose, BMI – all continuous)

Model 3 adjusted for all the variables in Model 2 plus depression symptoms (categorical), PWB (continuous), and existing illness (diabetes). For details see ‘Materials and methods’ and ‘Statistical analysis’

**Table 4:** Risk of a first event of CVD^a^ for lower cognitive function levels^b^ in women, Kaunas HAPIEE study, 2006–2016

Cognitive function	Cox models					
	Model 1		Model 2		Model 3	
	HR	95% CI	HR	95% CI	HR	95% CI
Immediate verbal recall sum	1.26	1.09–1.46	1.26	1.09–1.47	1.32	1.13–1.55
Delayed verbal recall	1.22	1.06–1.40	1.22	1.05–1.40	1.24	1.07–1.44
Semantic verbal fluency	1.15	0.99–1.33	1.12	0.97–1.31	1.16	0.99–1.36
Numerical ability	1.00	0.88–1.14	1.04	0.91–1.19	1.07	0.93–1.23
Cognitive speed and attention	1.11	0.97–1.28	1.10	0.95–1.28	1.08	0.92–1.26
Composite score of cognitive function	1.23	1.08–1.40	1.24	1.09–1.42	1.29	1.12–1.49

^a^ Individuals with existing CVD (IHD and stroke) at baseline survey were excluded, ^b^ per each 1 standard deviation decrease

*HAPIEE* Health, Alcohol and Psychosocial factors In Eastern Europe, *HR* hazard ratios, *CI* confidence interval

Model 1 adjusted for age (continuous variable), education and marital status (categorical variables)

Model 2 adjusted for all the variables in Model 1 plus lifestyle (smoking – categorical, physical activity in leisure (continuous – hours/week), alcohol consumption (continuous – drinks/week) and biologic factors (systolic or diastolic blood pressure – continuous, total cholesterol, HDL cholesterol, LDL cholesterol, triglycerides, fasting glucose, BMI – all continuous)

Model 3 adjusted for all the variables in Model 2 plus depression symptoms (categorical), PWB (continuous), and existing illness (diabetes). For details see ‘Materials and methods’ and ‘Statistical analysis’

After adjustment for socio-demographic factors (Model 1), a decrease per one SD in the scores of cognitive functions such as immediate verbal recall and delayed verbal recall, and the composite score of cognitive function significantly increased the risk of a first event of CVD in men (by 9–21%) and in women (by 22–26%). After additional adjustment for lifestyle and biological risk factors, depressive symptoms, PWB and existing illness (Model 2, Model 3), the risk of a first event of CVD remained statistically significant in men and women. However, in men and women, such a significant relationship was not determined for semantic verbal fluency, numerical ability, cognitive speed and attention.

## Discussion

The results of our study confirm that new CVD events are associated with a cognitive function composite score and a score of some specific domains. It is believed that cognitive functions may contribute a significant part towards the accumulation of cardiovascular risk factors [20], as cognitive decline is associated with hypertension, diabetes, obesity and dyslipidaemia [21]. A decreased cognitive function score is associated with cardiac dysfunction [22], meaning that one of the possible explanations for the association between CVD and lowered cognitive function might be a common cardiovascular risk factor.

Scientists suggested that common risk factors such as diabetes and hypertension might mediate the link between CVD and cognitive functions [16]. The previous study ascertained that a lower level of cognitive functions is associated with a higher incidence of cardiovascular events among the diabetes population [23]. Moreover, mortality due to recurrent heart failure might partly be affected by comorbidities during initial heart failure diagnosis, such as diabetes mellitus and depression [24].

Depressive symptoms were also an important predictor of CVD incidence in our study. A first CVD event was more frequent in participants who had lower PWB and depressive symptoms during the initial study. Symptoms of depression were previously linked with heart failure and mortality [25]. Particularly depressive symptoms are believed to explain the connection between cognitive decline and mortality [26]. Also, it is supposed that people with a lower cognitive function more often engage in unhealthy lifestyles, such as smoking, heavy alcohol consumption and physical inactivity [27]. Our study revealed that new CVD events are associated with smoking during the initial study. Alcohol consumption and physical inactivity were linked with new CVD events predominantly among women, and not significantly among men.

A lowered composite cognitive function score predicted the higher probability of a first event of CVD compared with normal cognitive function in men and women. Immediate and delayed verbal recall and the composite score of cognitive function significantly increased the risk of a first event of CVD after adjustment for sociodemographic, lifestyle and biological risk factors, as well as existing illnesses. However, a significant relationship was not determined for semantic verbal fluency, numerical ability, cognitive speed and attention. The difference might be due to different associations between cognitive functions and different cardiovascular illnesses. Previous studies ascertained that lowered cognitive function was a predictor of first and recurrent heart failure [16, 25], stroke [14, 28] and incident CVD [16]. However, coronary heart disease was only associated with a deterioration of cognitive function during the follow-up, but not with the initial level of cognitive function [16, 29]. Our previous study results also ascertained lower levels of cognitive functions after coronary heart disease [18].

### Study strengths and limitations

In the context of this study, some of its strengths should also be mentioned. The study examined and evaluated a relatively large sample of middle-aged and older urban residents representing a fairly diverse cross section of the population. During the study, the subjects were followed for a sufficiently long period of time. The study assessed the association of cognitive function with the incidence of a first case of cardiovascular disease. This association was established using a variety of models, controlling them for both sociodemographic and biological factors, and co-morbidities, some mental health and well-being factors and other lifestyle factors. This reduced a potential bias within the study, which may have been influenced by the factors listed above. In this prospective study, not only were the various components of cognitive function, such as the function of attention and cognition rate, direct and delayed memory capacity evaluated, but also their derived summative estimates. The study found that some cognitive functions were worse in both men and women who became ill or who died from a first event of CVD during the study period. The study revealed that a decline in cognitive function may be a good predictor of future morbidity or mortality from CVD, particularly among the male population.

The study also revealed several limitations that may have been relevant to the interpretation of the results obtained. First, the subjects may have developed other diseases, both acute and chronic, over the relatively long period of the study, which was associated with an increased incidence of CVD events or even death. Second, other genetic diseases, cases of physical disability and lifestyle-related factors that may predispose to cognitive impairment and be associated with a higher incidence of first CVD and death from other chronic diseases that were not measured and assessed in this study could not contribute to immeasurable misleading factors. Third, in our study the decrease in cognitive function during the follow-up was not controlled, because cognitive function was measured only at the beginning of the study. Fourth, the study also failed to assess some psychosocial factors in the subjects’ work environment that could have had some bearing on the higher incidence of first CVD events. And finally fifth, a lack of association between some specific lowered cognitive function domains at the baseline and the incidence of first events of CVD could be related to a low prevalence of such lowered cognitive functions and low indicators of the incidence of first events of CVD, especially among the women’s group.

## Conclusions

The findings of this follow-up study suggest that men and women with lower cognitive functions have an increased risk for a first event of CVD compared to participants with a higher level of cognitive functions.

## Data Availability

The datasets used and/or analysed during the current study are available from the corresponding author on reasonable request.

## Abbreviations

BMI: Body mass index
CI: Confidence interval
CVD: Cardiovascular diseases
HAPIEE: Health, Alcohol and Psychosocial Factors in Eastern Europe study
HDL: High-density lipoprotein
HR: Hazard ratios
ICD: International Classification of Diseases
IHD: Ischemic heart disease
LDL: Low-density lipoprotein
MONICA: Multinational Monitoring of Trends and Determinants in Cardiovascular Disease project
PWB: Psychological well-being
SD: Standard deviation
SE: Standard error
UK: United Kingdom
